# How to Scale Up Quality and Safety Program with the Home Care Accreditation

**DOI:** 10.5334/ijic.5698

**Published:** 2022-03-04

**Authors:** Laura Brunelli, Vittorio Cristofori, Claudio Battistella, Anna Paola Agnoletto, Anna Catelani, Cristina De Sarno, Bruna Odasmini, Simone Pauletto, Paola Stenico, Corrado Tosetto, Silvio Brusaferro

**Affiliations:** 1 Dipartimento di Area Medica, Università degli Studi di Udine, Udine, Italy; 2 SOC Accreditamento e Qualità, Azienda Sanitaria Universitaria Friuli Centrale, Udine, Italy; 3 Present address: Direzione Sanitaria, AULSS n.4 Veneto Orientale, Portogruaro, Italy; 4 Distretto di Cividale, Azienda Sanitaria Universitaria Friuli Centrale, Udine, Italy; 5 Servizio Sociale dei Comuni del Medio Friuli, ASP “Daniele Moro”, Codroipo, Italy; 6 Distretto di Tarcento, Azienda Sanitaria Universitaria Friuli Centrale, Udine, Italy; 7 Distretto 2 Alto Vicentino, AULSS7 Pedemontana, Thiene, Italy; 8 Servizio Territoriale Azienda Provinciale per i Servizi Sanitari di Trento, Trento, Italy; 9 Professioni Sanitarie della Riabilitazione, Azienda Sanitaria Universitaria Friuli Centrale, Udine, Italy

**Keywords:** primary care, accreditation: quality improvement, patient safety, healthcare quality improvement

## Abstract

**Introduction:**

The growing number of older people and the increasing burden of non-communicable diseases highlight the need for the integration between social and health services. To ensure high quality home care, common and consistent standards are essential. Our aim is to develop a validated accreditation tool for home care.

**Description:**

An integrated home care accreditation tool was developed including 26 standards and 144 items divided into six domains: Organization&Governance, Patient Safety&Risk Management, Professionals knowledge, Skills&Competences, Information&Communication, Care Integration, and Improvement&Innovation. Expert evaluation was conducted between August and November 2019; relevance and feasibility (RF) and expert agreement were analyzed.

**Discussion:**

A total of 21 experts participated in the validation process, with a response rate of 53%. A good RF score and agreement were obtained for 70% of the items and 65% of the standards. The best scores were obtained for *Individualized care project* (RF 8.4, agreement 100%), *Integrated care pathways* (RF 7.5, agreement 81%), *Access to the integrated health and social care system* (RF 8.1, agreement 86%), and *Multidimensional assessment of needs* (RF 8.1, agreement 86%).

**Conclusion:**

The existence of an integrated health and social care accreditation tool would help to improve the quality of home care, and make patients’ quality of life better and safer.

## Introduction

The growing elderly population is a common phenomenon throughout Europe [1,2], and the associated increase in the burden of non-communicable diseases have increased access to healthcare and social services in Italy [3,4]. The average age of the Italian population is expected to increase in the coming years, reaching 50 years in 2065, as the baby boomer cohort reaches 65 years and older [2]. Italian public healthcare on a regional basis guarantees universal health coverage, even if the quality of the services provided may vary from one region to another [5]. This heterogeneity is also a consequence of the 2001 constitutional reform, which entrusted the organization and management of healthcare services to Italian regions and autonomous provinces [6]. The Italian social service is provided to people in need by local municipalities according to social and economic conditions [7]. Therefore, the two services are managed separately and several factors hinder the integration process, such as the habit of the population to seek treatment in hospital [7], the scarce funding and the high specialization of both social services and primary care [8]. In addition, fragmentation and duplication of services contribute to inequality [9], suboptimal care, and higher costs [10]. Integrating services contributes to better access to and continuity of high-quality services, especially for frail older people and patients with chronic conditions and disabilities [10], and integrated care is likely to reduce costs and improve outcomes [11]. Several key issues are required to merge the two services, including political support, shared governance, stakeholder commitment, organizational change, national coordination, staff training, patient empowerment, improved funding and incentive mechanisms, shared health information and monitoring systems [12].

Home care, defined by WHO as care that aims to meet people’s health and social needs while they live in their homes [13], even with its heterogeneous implementation in different regions as provided in our national context, generally includes home medical consultation, biological sampling, nursing care, meal delivery, psychological support, administration of therapies and medications, health education of patient and caregivers, and empowerment for self-management of health-related equipment and technologies. In addition to these, as defined by the 1998 Italian Law No. 112, all services intended to remove and overcome situations of need and difficulty during the life-course e.g., social assistance for families in need, employment and residential services, are provided. Moreover, home care services work in collaboration with youth and family counseling centers, as well as mental health, drug addiction, and adolescent or adult disability services. Home care requires a high level of integration between social and health services, so common standards are essential to ensure quality home care. However, the relationships and coordination between professional and nonprofessional staff (e.g., family members, caregivers, neighbors, volunteers), the complexity of social care, and the existing gap in tools to evaluate the effectiveness of social services make identifying these common standards extremely difficult [14].

Accreditation of out-of-hospital health services can promote improvements in quality of care and patient safety, but nowadays there are few tools available to evaluate the performance of home care services. These tools are distinguishable according to the type of indicator used: outcome indicators -improvement or worsening of the clinical state or performance of the assisted patient- process indicators, and structural indicators. Specific tools may consider only one of them, e.g., HC-QIs – the interRAI’s second-generation home care quality indicator [15], or all three types of indicators, as OASIS – Outcome Assessment and Information Set [16] and HCQuAT – Home Care Quality Assessment Tool [17]. Even if some international experiences in the development and implementation of clinical assessment protocols have been accumulated by researchers and practitioners’ collaborative network, institutionalization of such experience as standard practice in Italy yet to occur. Even though Italian Law No. 328/00 requires it, there is no national accreditation system for social services, and each region can carry out the assessment independently. Regarding home care services, since 2015, when the first national accreditation model for out-of-hospital care services was developed by the National Agency for Regional Health Services (AGENAS) [18], it has been hardly been used by Italian regions. In some regions, such as Tuscany [19] and Emilia Romagna [20], other tools have been developed, but they failed to take into account the joint impact of health and social care at home level. Nevertheless, the application of the above-mentioned common standards in a home care accreditation would improve patient safety and the quality of care provided on behalf of the Italian National Healthcare Service, while promoting collaboration between professional and non-professional staff in a complex care setting.

The aim of the present study is to develop and validate an accreditation tool that includes health and social common standards for home care.

## Description of the methodology

### The home care accreditation tool development

In 2018 a multidisciplinary and multiprofessional research group consisting of a doctor, three nurses, a physiotherapist and two social workers coming from three northern Italian neighbouring regions critically reviewed the available AGENAS accreditation model for out-of-hospital care. First, a review on scientific and grey literature on the definition of home care and on the specific topic was conducted. Then, using the principles of the Deming cycle [21], in-depth group discussions were held based on the available literature and the researchers’ experience in home care until agreement was reached among them on the final version of a new accreditation tool for home care.

The developed tool proposal for home care accreditation included a set of 26 standards divided into six main domains: 1) Organization and governance (five standards) 2) Patient safety and risk management (five standards), 3) Professionals knowledge, skills and competences (four standards), 4) Information and communication (five standards), 5) Care integration (four standards), and 6) Improvement and innovation (three standards). The original set of standards and domains is shown in ***Table 1***; the full set including all items is available as supplementary material.

**Table 1:** Domains and standards of the home care accreditation tool proposal.


DOMAIN	STANDARD

**1.** Organization and governance	**1.1** Healthcare and social care planning and implementation **1.2** Healthcare network development **1.3** Responsibilities attribution **1.4** Quality of healthcare and social services evaluation **1.5** Prevention of service disruption and incident reporting management

**2.** Patient safety and risk management	**2.1** Building suitability and safe home access **2.2** Technical equipment management **2.3** Drug and food management **2.4** Risk assessment and management in care processes **2.5** Incident reporting system

**3.** Professionals knowledge, skills and competences	**3.1** Job description **3.2** Training program planning and evaluation **3.3** New employee training and support **3.4** Promoting self-care empowerment

**4.** Information and communication	**4.1** Information management **4.2** Clinical and social handover **4.3** Communication between care providers **4.4** Information and communication with citizens **4.5** Caregiver participation in care project

**5.** Care integration	**5.1** Access to the integrated health and social care system **5.2** Multidimensional assessment of needs **5.3** Individualized care project **5.4** Integrated care pathways

**6.** Improvement and innovation	**6.1** Improvement projects **6.2** New technology evaluation and implementation **6.3** Technical, professional and organizational innovation


Each standard contained a specific set of items developed according to the phases of the Deming cycle: plan, do, check, act. The total 144 items were distributed among the domains as follows: 28 for Domain 1, 36 for Domain 2, 17 for Domain 3, 27 for Domain 4, 19 for Domain 5, and 17 for Domain 6. For each item, the result of the evaluation of future compilers will be expressed in terms of met (M), partially met (PM), not met (NM), and not applicable (NA).

### The validation process

A group of 40 external experts in the field of primary care and social services was identified among professionals currently working in these services within northern Italian regions (namely Veneto and Friuli-Venezia Giulia). Professionals who had participated in the development of the home care accreditation tool (step 2.1) were excluded to avoid confirmation bias. Experts from these two Italian regions were selected based on the likelihood of their healthcare and social model and for reasons of convenience. Experts’ participation in the process of validation of the tool, conducted as a Delphi study, was voluntary and free of charge, and participation was not remunerated.

In August 2019, experts were invited by email to participate in the validation of the home care accreditation tool using the Delphi consensus method, following previous studies [22,23,24]; only publicly available email contacts were used. The aims and methods of the study were explained in detail; participants who returned their ratings and comments to the researchers were assumed to have given their implicit consent to participate in the study. Participants were reminded by e-mail and telephone before the survey closed in November 2019. Experts were asked to rate each item for relevance and feasibility (RF score) on a nine-point scale (1-minimum; 9-maximum). Specific suggestions or comments for improvement were also collected. Due to difficulties in experts recruitment and follow-up, the Delphi study had only one round. Agreement between experts’ ratings was calculated as a percentage. A good RF score was considered to be equal to or greater than 7; good agreement between experts was considered to exist if at least 70% of the expert ratings were greater than 7. When the agreement for an item was not reached, its inclusion for the future tool implementation could be carefully re-considered.

## Results

A total of 21 experts participated in the validation, with a response rate of 53%; 15 of them were medical directors of local health districts (71%) and six were managers of local social services (29%). We received 18 evaluations from the Friuli-Venezia Giulia region (86%) and three from the Veneto region (14%). The results of the assessment of domains, standards and single items are shown in ***Table 2***.

**Table 2:** The evaluation of domains, standards and single items expressed by mean RF score, standard deviation, mode of RF score, and agreement among experts.


DOMAIN/STANDARD/ITEM	MEAN	ST. DEV.	MODE	AGREEMENT

**1. ORGANIZATION AND GOVERNANCE**	7.6	1.2	8	76%

**1.1 Healthcare and social care planning and implementation**	7.6	1.1	8	86%

PLAN1	7.2	1.3	8	67%

PLAN2	7.8	0.9	8	90%

DO	8.0	0.8	8	95%

CHECK	7.7	1.0	8	86%

ACT	7.5	1.4	7	90%

**1.2 Healthcare network development**	7.7	1.2	8	76%

PLAN1	7.5	1.4	8	71%

PLAN2	7.8	1.2	8	76%

PLAN3	7.4	1.4	8	76%

PLAN4	7.8	1.2	9	86%

PLAN5	7.6	1.5	9	76%

DO	7.8	0.9	8	95%

CHECK	7.7	1.2	7	86%

ACT	8.0	0.8	7	100%

**1.3 Responsibilities attribution**	7.8	1.0	8	90%

PLAN1	8.2	0.8	9	100%

PLAN2	7.8	1.0	9	90%

DO	7.8	1.0	8	90%

CHECK	7.7	1.0	8	86%

ACT	7.5	0.9	8	86%

**1.4 Quality of healthcare and social services evaluation**	7.3	1.1	7	71%

PLAN	7.4	1.1	7	81%

DO	7.4	1.2	7	76%

CHECK	7.1	1.0	7	71%

ACT	7.2	1.2	8	71%

**1.5 Prevention of service disruption and incident reporting management**	7.4	1.4	8	71%

PLAN	7.1	1.8	9	71%

DO1	7.7	1.3	8	86%

DO2	7.0	1.3	8	76%

DO3	7.3	1.4	8	81%

CHECK	7.2	1.3	8	76%

ACT	7.7	1.4	9	76%

**2 PATIENT SAFETY AND RISK MANAGEMENT**	7.4	1.8	8	71%

**2.1 Building suitability and safe home access**	7.3	1.7	8	76%

PLAN1	7.9	1.5	8	90%

PLAN2	7.4	1.7	8	81%

DO1	7.7	1.5	8	90%

DO2	7.7	1.7	8	86%

CHECK	6.9	1.6	8	67%

ACT	6.6	1.9	8	62%

**2.2. Technical equipment management**	7.6	1.2	8	74%

PLAN1	7.4	1.1	8	74%

PLAN2	7.7	1.1	8	78%

DO1	7.8	1.1	9	84%

DO2	7.9	1.2	9	83%

DO3	7.9	1.2	8	84%

DO4	7.5	1.3	9	79%

CHECK	7.2	1.1	7	72%

ACT	7.2	1.2	6	67%

**2.3 Drug and food management**	6.7	2.2	9	50%

PLAN1	8.0	1.7	9	90%

PLAN2	7.1	2.5	9	63%

DO1	7.7	1.8	9	84%

DO2	6.2	2.3	9	45%

DO3	5.8	2.1	5	40%

DO4	6.1	2.5	9	50%

CHECK1	6.4	2.0	8	50%

CHECK2	6.4	2.0	8	50%

ACT	6.2	2.2	9	45%

**2.4 Risk assessment and management in care processes**	7.8	1.6	9	81%

PLAN1	8.3	1.2	9	95%

PLAN2	7.9	1.6	9	80%

DO1	7.8	1.2	8	90%

DO2	8.0	1.3	9	90%

DO3	7.7	1.3	8	90%

CHECK	7.4	2.0	8	86%

ACT	7.2	2.1	8	86%

**2.5 Incident reporting system**	7.7	1.6	8	81%

PLAN1	7.8	1.6	8	90%

PLAN2	7.9	1.6	8	95%

DO1	7.7	1.7	8	90%

DO2	7.6	1.7	8	86%

CHECK	7.5	1.7	8	81%

ACT	7.6	1.7	8	86%

**3 PROFESSIONALS KNOWLEDGE, SKILLS AND COMPETENCES**	7.0	1.7	7	48%

**3.1 Job description**	7.0	1.5	8	52%

PLAN	7.6	1.4	9	76%

DO	7.0	1.3	7	71%

CHECK	6.8	1.5	8	52%

ACT	6.5	1.6	6	43%

**3.2 Training program planning and evaluation**	7.4	1.6	9	67%

PLAN1	8.4	0.7	9	100%

PLAN2	8.2	0.8	9	100%

DO	6.7	2.1	9	52%

CHECK	7.0	1.5	7	62%

ACT	6.6	1.6	7	62%

**3.3 New employment training and support**	7.1	1.8	7	67%

PLAN	7.3	1.6	7	86%

DO	7.1	1.8	9	71%

CHECK	7.1	1.9	8	81%

ACT	6.7	1.9	7	62%

**3.4 Promoting self-care empowerment**	6.6	1.9	4	52%

PLAN	6.8	1.9	7	67%

DO	6.7	2.0	9	62%

CHECK	6.4	1.9	4	45%

ACT	6.5	1.9	4	57%

**4 INFORMATION AND COMMUNICATION**	7.5	1.5	8	76%

**4.1 Information management**	7.8	1.1	9	86%

PLAN1	7.1	1.4	6	57%

PLAN2	8.1	0.9	8	95%

DO1	7.9	1.1	9	90%

DO2	7.9	1.0	8	95%

DO3	8.1	0.8	8	100%

DO4	8.1	1.3	9	95%

DO5	8.0	0.9	8	95%

CHECK	7.6	1.0	7	90%

ACT	7.3	1.3	7	81%

**4.2 Clinical and social handover**	8.0	1.4	9	86%

PLAN	8.1	0.9	8	95%

DO1	7.9	2.0	9	86%

DO2	8.2	1.5	9	90%

CHECK	8.0	1.1	9	90%

ACT	7.9	1.2	9	86%

**4.3 Communication between care providers**	7.4	1.3	8	81%

PLAN	7.3	1.4	8	86%

DO	7.7	1.4	8	90%

CHECK	7.2	1.4	8	81%

ACT	7.3	1.4	8	85%

**4.4 Information and communication with citizens**	6.6	2.0	7	52%

PLAN	6.4	2.3	7	67%

DO1	7.0	1.7	7	67%

DO2	7.4	1.3	8	81%

CHECK	5.9	2.0	7	43%

ACT	6.0	2.1	7	48%

**4.5 Caregiver participation in care project**	7.8	1.5	8	86%

PLAN	8.5	0.7	9	100%

DO	8.0	1.1	9	90%

CHECK	7.6	1.7	8	90%

ACT	7.0	1.9	8	76%

**5 CARE INTEGRATION**	8.0	1.4	8	86%

**5.1 Access to the integrated health and social care system**	8.1	1.5	9	86%

PLAN	8.0	1.6	9	81%

DO	8.2	1.5	9	86%

CHECK	8.1	1.5	9	86%

ACT	8.0	1.5	9	86%

**5.2 Multidimensional assessment of needs**	8.1	1.3	9	86%

PLAN1	8.4	1.0	9	95%

PLAN2	8.2	1.4	9	86%

DO1	8.5	1.0	9	95%

DO2	8.1	1.4	9	86%

CHECK	7.6	1.6	8	76%

ACT	7.7	1.3	9	86%

**5.3 Individualized care project**	8.4	0.7	9	100%

PLAN	8.5	0.7	9	100%

DO	8.6	0.7	9	100%

CHECK	8.3	0.7	9	100%

ACT	8.3	0.7	9	100%

**5.4 Integrated care pathways**	7.5	1.6	9	81%

PLAN1	7.8	1.3	9	86%

PLAN2	7.4	1.7	8	81%

DO	7.9	1.0	8	90%

CHECK	7.1	1.8	7	81%

ACT	7.3	1.7	8	86%

**6 IMPROVEMENT AND INNOVATION**	6.5	1.6	7	53%

**6.1 Improvement projects**	6.7	1.2	7	55%

PLAN1	6.6	1.5	7	60%

PLAN2	7.0	1.1	8	60%

DO	6.8	1.2	7	65%

CHECK1	6.8	1.2	7	65%

CHECK2	6.6	1.2	7	58%

ACT	6.7	1.2	7	60%

**6.2 New technology evaluation and implementation**	6.4	1.6	7	48%

PLAN1	6.3	1.8	7	57%

PLAN2	6.5	1.6	5	52%

DO	6.4	1.7	7	52%

CHECK	6.4	1.6	7	52%

ACT	6.1	1.7	7	48%

**6.3 Technical, professional and organizational innovation**	6.4	1.9	6	43%

PLAN	6.9	1.3	6	48%

DO1	6.6	1.7	6	52%

DO2	6.6	1.7	6	48%

DO3	6.3	2.1	9	45%

CHECK	6.0	2.2	9	43%

ACT	6.2	2.1	9	43%


As defined in the section on the validation process, 101 of 144 items (70%) achieved a good RF score and agreement. Seventeen of 26 standards (65%) achieved good RF scores and agreement. The best scores were obtained for *Individualized care project* (RF 8.4, agreement 100%), *Integrated care pathways* (RF 7.5, agreement 81%), *Responsibilities attribution* (RF 7.8, agreement 90%), *Risk assessment and management in care processes* (RF 7.8, agreement 81%), *Information management* (RF 7.8, agreement 85%), *Caregiver participation in care project* (RF 7.8, agreement 86%), *Access to the integrated health and social care system* (RF 8.1, agreement 86%), and *Multidimensional assessment of needs* (RF 8.1, agreement 86%).

Worse scores were achieved by *Drug and food management* (RF 6.7, agreement 50%), *Information and communication with citizens* (RF 6.6, agreement 52%), *Improvement projects* (RF 6.7, agreement 55%), *New technology evaluation and implementation* (RF 6.4, agreement 48%), *Technical, professional and organizational innovation* (RF 6.4, agreement 43%). Five of the six domains achieved a good RF score experts RF and four of the six domains received a good agreement.

Of the experts’ comments (84), most related to Patient safety and risk management (38 comments), and Improvement and innovation domains (17 comments). Some comments related to typos in the original version, which were corrected. Other comments gave a flavor of the response each expert would have given if he/she had been asked to compile the tool as an end user, but at this stage this was not required. The comments provided insight into what difficulties might be encountered both in compiling the tool and in achieving the required standard, and will be eventually used to further revise the items.

## Discussion

The aim of this study was to develop and validate an integrated accreditation tool for home care as a first step towards improving the quality and patient safety of this service. The validation process resulted in a good assessment of the relevance and feasibility of our home care accreditation tool proposal, and a good level of agreement among experts, with these results confirming the accuracy of both the design and the contents of the tool. Nevertheless, themes where lower RF scores and lower levels of agreement were obtained (e.g., domain 6, Improvement and Innovation), seem to suggest that out-of-hospital health and social care is not yet prepared to deal with innovation and that these services may not be suited to implement a continuous improvement process. Considering that the ultimate aim of all accreditation models is to promote organizational improvement while improving quality and safety aspects, this finding reinforces our belief in the need for an accreditation tool that addresses these specific areas without excluding these domain. Although the accreditation tool developed by AGENAS [18] for out-of-hospital care and other tools developed in some Italian regions, such as Tuscany [19] and Emilia Romagna [20] are already available, the first one has hardly been used since 2015 and the latter two failed to take into account the joint impact of health and social care at home level. On the contrary, our proposal forces the organization to share strategies to plan, evaluate and improve their services. For example, we require health and social care services to identify a single point of contact for patients and their families where they can receive information, support and a multidimensional assessment of needs in a cost-effective way [11].

The Plan-Do-Check-Act model used in the development of the tool is one of its strengths, as it has been studied and effectively applied in other settings, such as clinical risk management in hospitals in the CARMINA project [25] to provide support for a cyclical review of all home care activities and their outcomes. Attention to clinical risk management is further reinforced in this tool by including standards for home safety and requiring organizations to provide patients with an incident reporting system to continuously monitor the occurrence of adverse events.

To ensure appropriate home care, both integration and deeper coordination between health and social care services is essential to deliver an equal, high quality, cost effective and person-centered service. In addition, effective collaboration between professional and non-professional staff from different backgrounds is required to optimize home care outcomes. Therefore, the presence of informal staff in home care is considered an added value in this tool, and therefore their participation in the care project is required. Moreover, the expert evaluations of our tool confirm the need for standardized communication processes to ensure proper clinical and social handover between care providers with different backgrounds (standard 4.2). The existence of an integrated health and social care tool would help improve the quality of care provided outside hospitals or by long-term care facilities, but also improve patients’ quality of life while reducing hospitalization rates [26]. Nevertheless, the active participation of patients in their care process contributes to their self-determination [27].

This study has some limitations: first, the experts involved in the validation process were from two northern and neighboring regions, which may reduce interregional variability in the way health and social services are delivered. Nevertheless, we consider the multidisciplinary and multiprofessional group that worked on the development of the tool to be a strength. Secondly, the original tool was developed and evaluated in the original language (Italian), so a direct comparison with other authors is not possible. However, we believe that the specificity of a single nation in terms of value system and language was worth having its own tool. Nevertheless, the translation has been reproduced to facilitate the discussion.

## Conclusion

In conclusion, to the best of our knowledge, this is the first Italian integrated accreditation tool for home care, and we believe that it should be considered at the national level, with the ultimate goal of ensuring provision of the essential levels of care required at the national level.

## Additional File

The additional file for this article can be found as follows:

10.5334/ijic.5698.s1 Supplementary Material. The full set of domains, standards and items of the home care accreditation tool.
