# Supplementary material for: How to Scale Up Quality and Safety Program with the Home Care Accreditation
Source: Int J Integr Care. 2022 Mar 4;22(1):19. doi: 10.5334/ijic.5698 (PMC8896250; doi:10.5334/ijic.5698)
Supplement: Supplementary Material. — The full set of domains, standards and items of the home care accreditation tool. [file ijic-22-1-5698-s1.pdf]

Domain	Standard	Item	
1. Organization and governance	1.1 Healthcare and social care planning and implementation	PLAN1	The organization adopted a strategic plan which aims are based on local need assessment, and are coherent with the organization’s vision, mission, and engage all stakeholders of the integrated services system
		PLAN2	The organization adopted an annual activity, quality and safety plan, where aims, actions and time scheduling are identified
		DO	The implementation of the actions planned is documented
		CHECK	The fulfilling of both strategic and annual activity plans is periodically evaluated
		ACT	The results of performance monitoring are made available and used for revision and improvement purposes
	1.2 Healthcare network development	PLAN1	The organization formally defined:<br>- the relationship among all stakeholders of the integrated services system, including hospitals and other healthcare facilities
		PLAN2	- the integrated clinical pathways for main chronic diseases (including palliative care for adults and children)
		PLAN3	- the inclusion of all stakeholders of the integrated services system
		PLAN4	- roles and responsibilities
		PLAN5	- outcome indicators
		DO	Healthcare pathways are adopted ensuring continuity with home care
		CHECK	The organization annually monitors of the implementation of care pathways and defined actions
		ACT	The results of the performance monitoring are made available and used for revision and improvement purposes
	1.3 Responsibilities attribution	PLAN1	The organization adopted a document including:<br>- the organizational chart, processes and responsibilities attribution, power of attorney
		PLAN2	- criteria set for professional evaluation
		DO	The organization implements contents of this document on responsibilities attribution
		CHECK	The organization monitor the implementation of the contents of this document
		ACT	There is a system in place to identify important changes in the organization or provision of services, and this information is used for revision and improvement purposes
	1.4 Quality of healthcare and social services evaluation	PLAN	The organization defined the processes of quality of services assessment, the processes of responsibility attribution, the identification of required standards, professionals and citizens participation, results sharing
		DO	There is evidence of the quality plan implementation
		CHECK	There is evidence of the quality plan assessment and stakeholder engagement
		ACT	The results of the monitoring are reported to stakeholders and are used for revision and improvement purposes
	1.5 Prevention of service disruption and incident reporting management	PLAN	The organization along with citizens’ delegates defines the policy for prevention and management of service disruption/complaint
		DO1	There is evidence of the implementation of the policy for prevention and management of service disruption/complaint
		DO2	The organization implements actions for sustaining citizens’ feedback and for assessing customer

			satisfaction
		DO3	The analysis processes and possible service disruption is documented
		CHECK	Citizens' feedbacks, customer satisfaction and processes analyses are analyzed in-depth and results are discussed with stakeholders
		ACT	The results of the monitoring are used for revision and improvement purposes
2 Patient safety and risk management	2.1 Building suitability and safe home access	PLAN1	The organization adopted an environmental risk management plan to identify risks for users and professionals, intervention to be implemented and related indicators
		PLAN2	The organization defined an intervention plan for risk management and promotion of home safety culture involving all stakeholders
		DO1	There is evidence of the implementation of:<br>- intervention for prevention of environmental risk
		DO2	- education about and involvement of citizens and users in risk management
		CHECK	There is evidence of at least one annual monitoring of environmental risk management indicators and safety promotion intervention
		ACT	The results of the monitoring are reported to professionals and citizens and are used for both processes and education revision and improvement purposes
	2.2 Technical equipment management	PLAN1	The organization adopted a plan for technical equipment management and maintenance
		PLAN2	The adopted plan includes at least:<br>- the procedure for the home management of emergencies due to technical equipment disruption,<br>- time scheduling and methodology for educational need assessment among professionals
		DO1	An inventory is available and includes all technical equipment
		DO2	Technical documentation and user manuals are available for all technical equipment. Instructions for management life-saving technical equipment disruption are also available
		DO3	Maintenance and periodic revision records for all technical equipment is available
		DO4	There is evidence of education conducted on the issues resulting from the need assessment
		CHECK	Indicators for the technical equipment management are periodically monitored
		ACT	The results of the monitoring activity are used for revision and improvement purposes for technical equipment management and purchase
	2.3 Drug and food management	PLAN1	The organization defined drug and food management procedures, including all aspects from prescription to disposal. Minimum contents include:<br>- aims and minimum safety level for home drug management<br>- roles and responsibilities for all stakeholders<br>- management of possible problems arising during and after drug administration<br>- reasons and methods for medication reconciliation<br>- read-back method for phone drug prescription<br>- indicators
		PLAN2	There is a specific procedure for high-risk (e.g. insulin, anticoagulant, blood transfusion, chemotherapy, terminal sedation) and look-alike-sound-alike (LASA) drug management
		DO1	There is evidence in home records of the implementation of correct drug and food management procedures
		DO2	There is evidence of:

			- citizen/user/caregiver health literacy level<br>- educational planning including feedback from recipients (e.g. teach-back)
		DO3	There is evidence in home records of drug periodical update, this process involving all stakeholders who perform drug therapy management and medication reconciliation
		DO4	There is evidence of actions implemented to avoid drug management errors (including those for LASA drugs)
		CHECK1	The organization monitor procedures' indicators
		CHECK2	The organization monitor drug and food management procedures for every single patient
		ACT	The results of the monitoring are shared with all stakeholders and used for revision and improvement purposes
	2.4 Risk assessment and management in care processes	PLAN1	The organization adopted specific procedures for the risk assessment and management about:<br>- bundle adherence<br>- patient identification, falls, malnutritional and functional decay, pressure ulcers, infections, pain<br>- biological exposure risk and manual handling for loads for professionals
		PLAN2	Have been identified:<br>- criteria/tools for high-risk patient identification<br>- identification criteria for patient risk exposure during healthcare<br>- risk management and information sharing with users/professionals<br>- tools for risk management<br>- educational pathways for users/professionals<br>- interventions to be implemented<br>- time schedule for re-evaluation<br>- incident reporting and management procedure<br>- outcome indicators
		DO1	The implementation of risk assessment and management in care process by all stakeholders is documented. The results of the risk assessment and intervention planning is documented in clinical records and within the individualized care pathway
		DO2	Professionals education, as well as patient/caregiver empowerment, are documented
		DO3	Technical equipment, information and tools for critical situation management (fall/danger/pressure ulcers) are available at home
		CHECK	The organization periodically evaluates the risk assessment and management procedures, throughout data collection and active surveillance
		ACT	The results of this monitoring are used for revision and improvement purposes
	2.5 Incident reporting system	PLAN1	The organization adopted a shared procedure for incident reporting from all stakeholders, with at least:<br>- identified reporting tool for anonymous data collection and management<br>- systematic internal (professionals) and external communication (users, caregivers, citizens' organizations) tool
		PLAN2	The organization adopted a management procedure for sentinel events
		DO1	There is evidence of the procedure being activation when the case (adverse event, near miss, sentinel event)
		DO2	Incident reporting data are completely and promptly available

		CHECK	Incident reporting data are deeply discussed among stakeholders
		ACT	Incident reporting data are used for revision and improvement purposes
3. Professionals knowledge, skills and competences	3.1 Job description	PLAN	The organization adopted a formal job description describing, roles, responsibilities and privileges
		DO	Implementation and updating of the formal job description throughout the organization is documented
		CHECK	Job description implementation and updating is assessed
		ACT	The results of the job description monitoring are used for revision and improvement purposes
	3.2 Training program planning and evaluation	PLAN1	The organization adopted an annual educational plan with the contribution of professionals, the responsibility of its fulfillment has been identified
		PLAN2	The educational plan includes educational need assessment, planning priority criteria identification and professional profile set of required competences
		DO	In accordance with the educational plan, there is an educational dossier with acquired competences and habilitations for each professional
		CHECK	The acquisition of new competences as required by the educational plan is monitored by the organization
		ACT	The results of the monitoring are used for education revision and improvement purposes
	3.3 New employee training and support	PLAN	The organization adopted a plan for the new employee training and support, including welcome, competences and attitudes assessment, education regarding clinical risk, health at work, and specific home care competences; a tutor and time schedule are identified for each new employee evaluation
		DO	The implementation of the plan for new employee training and support, as well as results of competences evaluation and end of trial period are documented
		CHECK	According to professional role, performance and aims fulfillment are evaluated
		ACT	The results of the evaluation of the new employee training and support program and of professional performance are used for revision and improvement purposes
	3.4 Promoting self-care empowerment	PLAN	The organization defined an educational program for professionals in order to enable them to support citizens and promote their empowerment
		DO	The availability of educational programs for professionals with regards to educational therapy is documented
		CHECK	Knowledge and competence acquisition of professionals performing educational therapy are monitored
		ACT	The results in terms of educational programs effectiveness are used for revision and improvement purposes
4. Information and communication	4.1 Information management	PLAN1	The organization adopted a data management policy based on citizens and their organization needs
		PLAN2	The organization defined data access and management process, according to privacy and safety policies
		DO1	The organization defined the process of information management ensuring:<br>- information traceability, data management and transmission
		DO2	- data collection for activity planning and monitoring
		DO3	- data sharing with national or regional level institution
		DO4	- accessible, prompt and transparent information for all stakeholders
		DO5	- professionals' education on information correct management procedures
		CHECK	The organization monitors information management implementation and related professionals' competences, including quality of information collected in terms of reliability, accuracy, usefulness in standard reports

		ACT	The results of the monitoring activity are used to update information databases and reports
	4.2 Clinical and social handover	PLAN	The organization adopted a handover plan including both oral and written methods to ensure the continuity of care
		DO1	A home integrated clinical record with the engagement of all stakeholders is available; this tool ensures clinical monitoring and continuity of care
		DO2	Integrated handover tools, both for oral and written communications, are available
		CHECK	Quality of home integrated clinical record is evaluated, and audit are conducted in case of need
		ACT	The results of home integrated clinical record are used for revision and improvement purposes
	4.3 Communication between care providers	PLAN	The organization adopted a communication policy, both for internal and external use
		DO	Internal and external information sharing, including professionals' and working groups meetings, are documented
		CHECK	The organization monitor the effectiveness of internal and external communication
		ACT	The results of the monitoring activity are used for communication revision and improvement purposes
	4.4 Information and communication with citizens	PLAN	The organization adopted a plan for information and communication with citizens
		DO1	The organization provides and regularly update a document illustrating all available care services and their quality of standard; the contribution of professionals, citizens, voluntary organizations are required
		DO2	Informative material on home integrated care services is available in different languages, according to local presence of cultural/language groups
		CHECK	Citizens' awareness of available home integrated care services is regularly assessed in collaboration with citizens' organizations
		ACT	The results of these awareness assessment are used for revision and improvement purposes
	4.5 Caregiver participation in care project	PLAN	The organization defined the procedure for the inclusion of the patient/caregiver in the home care integrated project, which includes:<br>- informed consent, when requested by law<br>- individualized care pathway sharing and patient choice of the level of engagement<br>- responsibility attribution for planned activities
		DO	Patient/caregiver empowerment and participation within their home care integrated project is documented
		CHECK	The level of patient/caregiver empowerment and participation within the home care integrated project is assessed
		ACT	The results of the empowerment and participation assessment are shared with patient/caregivers and are used for the home care integrated project revision and improvement purposes
5. Care integration	5.1 Access to the integrated health and social care system	PLAN	The organization identified integrated services access point for citizens with social, health and mixed needs
		DO	Tools for patient need assessment are used in each integrated services access point
		CHECK	The integrated services access point activity is monitored (e.g. access number, activities conducted, need assessment)
		ACT	The results of this monitoring are used for revision and improvement purposes
	5.2 Multidimensional assessment of needs	PLAN1	The organization defined the multidimensional need assessment unit
		PLAN2	Specific tools for the multidimensional assessment of need have been identified
		DO1	The implementation of the multidimensional need assessment using the defined tools is documented

		DO2	The multidimensional need assessment is performed using validated tools
		CHECK	The organization monitors the implementation of multidimensional need assessment in terms of used tools and composition of the multidisciplinary group
		ACT	The results of the monitoring are used for revision and improvement purposes
	5.3 Individualized care project	PLAN	The organization defined minimum contents of the individualized care pathway in terms of actions, responsibilities, objectives, time schedule, resources needed and case manager identification
		DO	Patient record includes the individualized care pathway, defined in agreement with patient/caregiver
		CHECK	The individualized care pathway implementation and the completeness of records is monitored
		ACT	The results of the monitoring are used for revision and improvement purposes
	5.4 Integrated care pathways	PLAN1	The organization defined an integrated care pathway for frail patients
		PLAN2	This pathway includes at least actions and related responsibilities identification
		DO	The implementation of the integrated care pathway is documented
		CHECK	The organization monitors the adherence to the integrated care pathway throughout indicators and standards
		ACT	The results of the monitoring are used for revision and improvement purposes
6. Improvement and innovation	6.1 Improvement projects	PLAN1	The organization adopted a strategic quality improvement plan, which include aspects related to clinical risk management; this plan has been shared with stakeholders
		PLAN2	The organization set priorities and defined actions and objectives within the quality improvement plan. Minimum elements are specific objectives, activities time scheduling, responsibilities, resources, outcome indicators
		DO	The implementation of the quality improvement plan and its priority activities is documented
		CHECK1	The assessment of the indicators of the quality improvement plan is documented
		CHECK2	The quality improvement plan outcomes are reported to stakeholders
		ACT	The results of the quality improvement plan monitoring are used for revision and improvement purposes
	6.2 New technology evaluation and implementation	PLAN	The organization defined new technologies assessment, identification, purchasing and placement procedures
		DO1	The technologies need assessment and its evaluation in terms of priority of purchasing is documented
		DO2	New technologies assessment, identification, purchasing and placement procedures according to set priority is documented
		CHECK	The organization monitors the new technologies assessment, identification, purchasing and placement procedures
		ACT	The results of the monitoring are used for revision and improvement purposes
	6.3 Technical, professional and organizational innovation	PLAN	The organization defined technical, professional and organizational innovation assessment
		DO1	The technical, professional and organizational innovation need assessment and its evaluation in terms of priority of purchasing is documented
		DO2	New technical, professional and organizational innovation priority plan is documented
		DO3	The engagement of professionals and citizens in the new technical, professional and organizational innovation acquisition is documented
